# Chronic kidney diseases in mixed ancestry south African populations: prevalence, determinants and concordance between kidney function estimators

**DOI:** 10.1186/1471-2369-14-75

**Published:** 2013-04-02

**Authors:** Tandi E Matsha, Yandiswa Y Yako, Megan A Rensburg, Mogamat S Hassan, Andre P Kengne, Rajiv T Erasmus

**Affiliations:** 1 Department of Biomedical Sciences, Faculty of Health and Wellness Science, Cape Peninsula University of Technology, Cape Town, South Africa; 2 Division of Chemical Pathology, Faculty of Medicine and Health Sciences, National Health Laboratory Service (NHLS) and University of Stellenbosch, Cape Town, South Africa; 3 Department of Nursing and Radiography, Faculty of Health and Wellness Science, Cape Peninsula University of Technology, Cape Town, South Africa; 4 NCRP for Cardiovascular and Metabolic Diseases, South African Medical Research Council, Cape Town, South Africa

**Keywords:** CKD-EPI eGFR, Cockroft-Gault eGFR, MDRD eGFR, Prevalence, South Africa

## Abstract

**Background:**

Population-based data on the burden of chronic kidney disease (CKD) in sub-Saharan Africa is still very limited. We assessed the prevalence and determinants of CKD, and evaluated the concordance of commonly advocated estimators of glomerular filtration rate (eGFR) in a mixed ancestry population from South Africa.

**Methods:**

Participants were a population-based sample of adults selected from the Bellville-South community in the metropolitan city of Cape Town. eGFR was based on the Cockroft-Gault (CG), Modification of Diet in Kidney Disease (MDRD) and CKD Epidemiology Collaboration (CKD-EPI) equations (with and without adjustment for ethnicity). Kidney function staging used the Kidney Disease Outcome Quality Initiative (KDOQI) classification. Logistic regressions and kappa statistic were used to investigate determinants of CKD and assess the agreement between different estimators.

**Results:**

The crude prevalence of CKD stage 3–5 was 14.8% for Cockcroft-Gault, 7.6% and 23.9% respectively for the MDRD with and without ethnicity correction, and 7.4% and 17.3% for the CKD-EPI equations with and without ethnicity correction. The highest agreement between GFR estimators was between MDRD and CKD-EPI equations, both with ethnicity correction, Kappa 0.91 (95% CI: 0.86-0.95), correlation coefficient 0.95 (95% CI: 0.94-0.96). In multivariable logistic regression models, sex, age and known hypertension were consistently associated with CKD stage 3–5 across the 5 estimators.

**Conclusions:**

The prevalence of CKD stages greater than 3 is the highest reported in Africa. This study provides evidence for support of the CKD-EPI equation for eGFR reporting and CKD classification.

## Background

Chronic kidney disease (CKD) has become a major public health problem worldwide, and this has necessitated the development of guidelines for the definition and classification of the disease by the Kidney Disease Outcomes Quality Initiative (KDOQI) [1,2]. It has been estimated that approximately 500 million individuals globally have CKD, a number that translates to about 1 in 10 affected adults [3]. The terminal stage of CKD, end stage renal disease (ESRD), requires renal replacement therapy or kidney transplant at enormous cost to individuals and national health budgets. It is estimated that over 1.4 million people worldwide receive renal replacement therapy to prolong life, with the incidence increasing by 8% annually [4,5]. Furthermore, the presence of CKD is associated with premature mortality from cardiovascular diseases (CVD). The risk of cardiac death is increased by 46% in people with a glomerular filtration rate (GFR) between 30 and 60 ml/min per 1.73 m^2^ independent of traditional cardiovascular risk factors (including diabetes and hypertension) [6]. The magnitude of CKD in Sub-Saharan Africa (SSA) countries is still poorly characterized, although there are speculations that the incidence rates are 3–4 times higher than those in developed countries [7]. In general, available data on CKD in Africa have been largely derived from hospital-based studies conducted in tertiary health care facilities and very few from community-based studies [8,9]. Some of the studies reported on the performance of the GFR estimating equations rather than the prevalence of CKD [10-12].

The reported high fatality rate associated with CKD in Africa has been attributed to a number of factors including the increasing prevalence of infectious diseases, late referrals of individuals with CKD to specialists, poor prognosis, limited renal replacement therapy, and the lack of agreement on the definition of CKD and standardisation of tests that are currently used for diagnosing the disease [10-13]. With specific reference to CKD diagnosis, the three commonly used estimators of GFR include the Cockcroft-Gault, the Modification of Diet in Renal Disease (MDRD) and the CKD Epidemiology Collaboration (CKD-EPI) equations, and each diagnoses a different subgroup of patients with CKD [11,12,14,15]. The more acclaimed MRDR and CKD-EPI equations apply a correction factor for ethnicity, which may substantially affect the diagnosis of CKD without being necessarily valid in all settings [11,12]. Therefore, in addition to improving the knowledge base on the magnitude of CKD at the population level in Africa, efforts are needed to improve the accuracy of the diagnosis of the disease. The aim of the present study was to assess the magnitude and determinants of CKD in a community based cohort, and evaluate the agreement between commonly advocated kidney function estimators in a mixed ancestry African population.

## Methods

### Study setting and population

Study participants were members of a cohort study conducted in Bellville South, a mixed ancestry township formed in the late 1950s, and which is located in the metropolitan city of Cape Town, South Africa. The mixed ancestry is a South African population group comprising 32-43% Khoisan, 20–36% Bantu-speaking African, 21 – 28% European and 9 – 11% Asian ancestry [16]. The study setting, survey design and procedures have been described in details elsewhere [17,18]. Briefly, eligible participants were invited to take part in a community based survey from January 2008 to March 2009 (Cohort 1), and January 2011 to November 2011 (Cohort 2). The study was approved by the Cape Peninsula University of Technology, Faculty of Health and Wellness Sciences ethics committee (Reference Number: CPUT/HW-REC 2008/002 and CPUT/HW-REC 2010). The study was conducted according to the Code of Ethics of the World Medical Association (Declaration of Helsinki). All participants signed written informed consent after all the procedures were fully explained in the language of their choice.

### Clinical data

All consenting participants received a standardized interview and physical examination during which blood pressure was measured according to the World Health Organisation (WHO) guidelines [19] using a semi-automatic digital blood pressure monitor (Rossmax PA, USA) on the right arm in a sitting position. Other clinical measurements included the body weight, height, waist and hip circumferences. Weight (to the nearest 0.1 kg) was determined in a subject wearing light clothing and without shoes and socks, using a Sunbeam EB710 digital bathroom scale, which was calibrated and standardized using a weight of known mass. Waist circumference was measured using a non-elastic tape at the level of the narrowest part of the torso, as seen from the anterior view. All anthropometric measurements were performed three times and their average used for analysis. Participants with no history of doctor diagnosed diabetes mellitus underwent a 75 g oral glucose tolerance test (OGTT) as recommended by the WHO [20].

### Laboratory measurements

Blood samples were collected after an overnight fast and processed for further biochemical analysis. Plasma glucose was measured by enzymatic hexokinase method (Cobas 6000, Roche Diagnostics, Germany) and glycated haemoglobin (HbA1c) by turbidimetric inhibition immunoassay (Cobas 6000, Roche Diagnostics, Germany) this being a National Glycohaemoglobin Standardisation Programme (NGSP) certified method. Creatinine levels were measured using the standardized creatinine assay (Cobas 6000, Roche Diagnostics, Germany).

### Definitions and calculations

Diabetes status was based on a history of doctor-diagnosis, a fasting plasma glucose >=7.0 mmol/l and/or a 2-hour post-OGTT plasma glucose >11.1 mmol/l. Hypertension was based on a history of doctor diagnosed hypertension and/or receiving medications for hypertension or average systolic blood pressure ≥140 mmHg and/or average diastolic blood pressure ≥90 mmHg. Kidney function was assessed through estimated glomerular filtration rate (eGFR) for which both the Cockcroft-Gault equation [21] (with correction for the body surface area using the formula by Du Bois) [22], 4-variable Modification of Diet in Renal Disease (MDRD) equation [23,24] applicable to standardised serum creatinine values, and the Chronic Kidney Disease Epidemiology Collaboration (CKD-EPI) equation [25] were used, with and without ethnicity adjustment for all participants. Staging of kidney function was based on the National Kidney Foundation Disease Outcomes Quality Initiative (NKF-KDOQI) classification [26]. An eGFR<60 ml/min was used to define chronic kidney disease (or CKD stage 3–5).

### Statistical analysis

The R statistical software version 2.13.0 (13-04-2011), (The R Foundation for Statistical Computing, Vienna, Austria) was used for data analysis. General characteristics of the study groups are summarized as count and percentage for qualitative variable, mean and standard deviation (SD) or median and 25^th^-75^th^ percentiles for quantitative variables. Group comparisons used chi square tests and equivalents for qualitative variables, and Student’s t-test and non-parametric equivalents for quantitative variables. Agreement between kidney function estimators was assessed on the continuous scale with the use of the Spearman’s correlation, and across categories of estimated kidney function with the use of the kappa statistic. The age standardized prevalence of CKD was calculated in ten-year-intervals using the standard world population distribution as projected by the WHO for 2000–2025 [27]. The direct standardization was applied. Predictors of CKD were investigated with the use of logistic regression models. A p-value <0.05 was used to characterise statistically significant results.

## Results

### Baseline characteristic of the study population

The initial study sample comprised 1256 subjects, 54 of whom had missing data on serum creatinine or any of the variables included in the estimation of kidney function (age, sex or weight) and were therefore excluded. The final sample included 1202 subjects of whom905 (75.3%) were females. The general characteristics of the study population are summarised in Table 1. The mean age was 52.9 years, with a borderline difference between men and women (54.2 vs. 52.4 years, p=0.06). With the exception of fasting plasma glucose (p=0.55), HbA1c (p=0.75), triglycerides (p=0.17), serum cotinine (p=0.05) and prevalent diabetes (p=0.80), significant differences were observed between men and women with regard to several baseline characteristics.

**Table 1:** Clinical characteristics of the study population overall and by sex

**Variables**	**Male**	**Female**	**p-value**	**overall**
N	297	905		1202
Mean age, year (SD)	54.2 (15.4)	52.4 (14.6)	0.06	52.9 (14.8)
Mean body mass index, kg/m^2^ (SD)	26.6 (6.4)	31.0 (7.1)	<0.0001	29.9 (7.2)
Mean waist circumference, cm (SD)	94 (15)	97 (15)	0.002	96 (15)
Mean hip circumference, cm (SD)	101 (11)	112 (14)	<0.0001	109 (14)
Mean waist-to-hip ratio, (SD)	0.93 (0.08)	0.87 (0.09)	<0.0001	0.88 (0.09)
Mean systolic blood pressure, mmHg (SD)	128 (19)	124 (20)	0.0006	125 (20)
Mean diastolic blood pressure, mmHg (SD)	78 (14)	75 (12)	0.001	76 (13)
Mean fasting blood glucose, mmol/l (SD)	6.4 (3.4)	6.3 (2.6)	0.55	6.3 (3.0)
Mean 2-hour blood glucose, mmol/l (SD)	6.8 (3.0)	7.5 (3.6)	0.001	7.4 (3.5)
Mean HbA1c,% (SD)	6.3 (1.6)	6.3 (1.4)	0.75	6.3 (1.4)
Mean total cholesterol, mmol/l (SD)	5.3 (1.1)	5.6 (1.2)	0.0001	5.6 (1.2)
Mean triglycerides, mmol/l (SD)	1.5 (0.9)	1.5 (0.9)	0.17	1.5 (0.9)
Mean HDL cholesterol, mmol/l (SD)	1.2 (0.4)	1.3 (0.3)	0.0001	1.3 (0.4)
Mean LDL cholesterol, mmol/l (SD)	3.4 (1.0)	3.7 (1.0)	0.0006	3.6 (1.0)
Median serum cotinine, (25^th^-75^th^ percentiles)	10 (10–311)	10 (9–378)	0.05	10 (9–284)
Median GGT, (25^th^-75^th^ percentiles)	32 (23–49)	25 (18–39)	<0.0001	27 (19–42)
Any diabetes, n (%)	80 (26.9)	237 (26.2)	0.80	317 (26.4%)
Current smoking, n (%)	146 (49.2)	341 (37.7)	0.0005	487 (40.5)
Estimated glomerular filtration rate (eGFR), ml/min				
Cockcroft-Gault, mean (SD)	84.1 (27.0)	94.2 (33.7)	<0.0001	91.7 (32.5)
Cockcroft-Gault <60, n (%)	57 (19.2)	121 (13.4)	0.014	178 (14.8)
4-v-MDRD, mean (SD)	102.8 (26.5)	95.4 (28.0)	<0.0001	97.3 (27.8)
4-v-MDRD <60, n (%)	15 (5.0)	77 (8.5)	0.05	92 (7.6)
4-v-MDRD without ethnicity correction, mean (SD)	79.9 (20.6)	74.2 (21.8)	<0.0001	75.6 (21.6)
4-v-MDRD without ethnicity correction <60, n (%)	45 (15.1)	242 (26.7)	<0.0001	287 (23.9)
CKD-EPI, mean (SD)	97.2 (23.0)	93.4 (25.7)	0.02	94.3 (25.1)
CKD-EPI <60, n (%)	17 (5.7)	72 (8.0)	0.20	89 (7.4)
CKD-EPI without ethnicity correction, mean (SD)	83.9 (19.9)	80.6 (22.1)	0.02	81.4 (21.6)
CKD-EPI without ethnicity correction <60, n (%)	38 (12.8)	170 (18.8)	0.02	208 (17.3)

***Abbreviations*:** CKD-EPI, Chronic Kidney Disease Collaboration Epidemiology; GGT, gamma glutamyltransferase; HbA1c, glycosylated haemoglobin A1c; HDL-C, high density lipoprotein-cholesterol; LDL-C, low density lipoprotein-cholesterol; 4-v-MDRD, four variable Modification of Diet in Renal Disease; SD, standard deviation.

### Correlation between eGFR as calculated by cockcroft-gault, MDRD, and CKD-EPI equations

**Figure 1:**
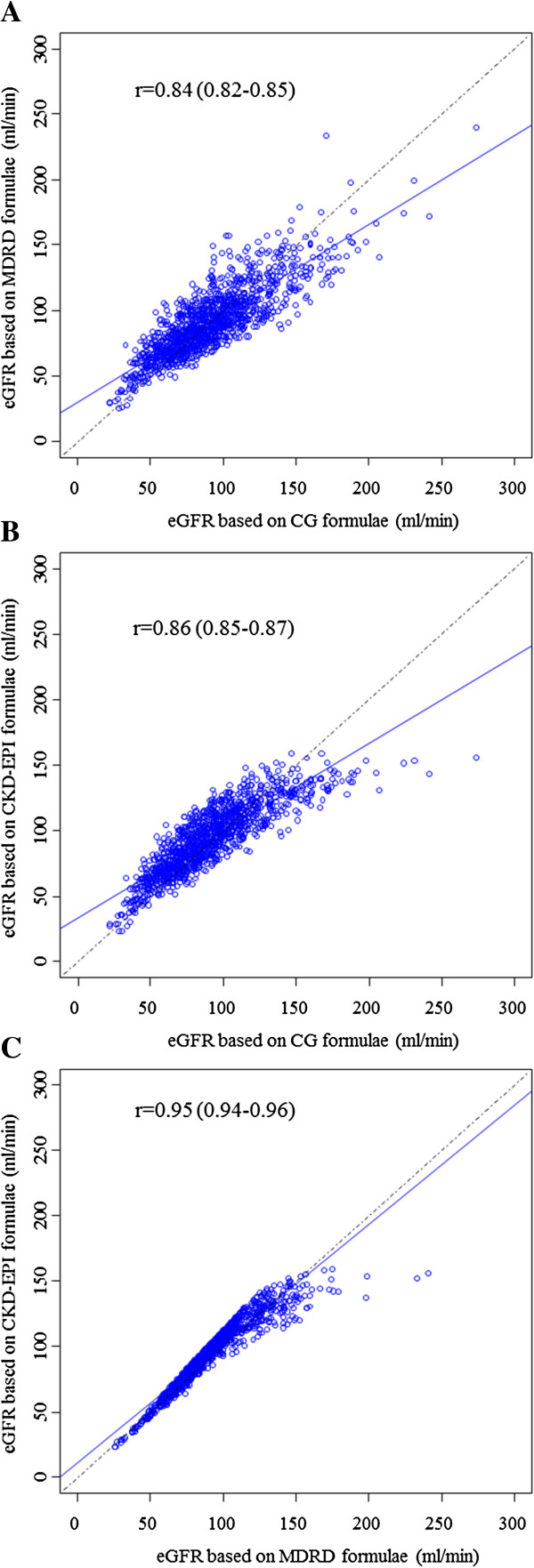
**Spearman’s correlation for the three GFR estimating equations. A**) four variable Modification of Diet in Renal Disease (4-v-MDRD) vs Cockcroft-Gault (CG), **B**) Chronic Kidney Disease Collaboration Epidemiology (CKD-EPI) vs CG and **C**) CKD-EPI vs 4-v-MDRD.

The mean estimated glomerular filtration rate (eGFR) was 91.7 ml/min (Cockcrof-Gault), 97.3 ml/min (MDRD with ethnicity correction), 75.6 ml/min (MDRD without ethnicity correction), 94.3 ml/min (CKD-EPI with ethnicity correction) and 81.4 ml/min (CKD-EPI without ethnicity correction) (Table 1). Compared with men, eGFR in women was higher based on Cockcroft-Gault estimator, and lower based on all other estimators (all p≤0.02). There was a positive and significant correlation between different estimates of GFR. The Spearson’s correlation coefficient (95% confidence interval) was 0.84 (0.82-0.85) for Cockcroft-Gault vs. MDRD (with or without ethnicity correction), 0.86 (0.85-0.87) for Cockcroft-Gault vs. CKD-EPI (with or without ethnicity correction) and 0.95 (0.94-0.96) MDRD vs. CKD-EPI (Figure 1). Equivalent results were 0.86 (0.82-0.88), 0.70 (0.63-0.75) and 0.94 (0.92-0.95) in men and, 0.88 (0.87-0.90), 0.76 (0.73-0.79) and 0.96 (0.95-0.96) in women.

### Staging of kidney function by different estimators

**Figure 2:**
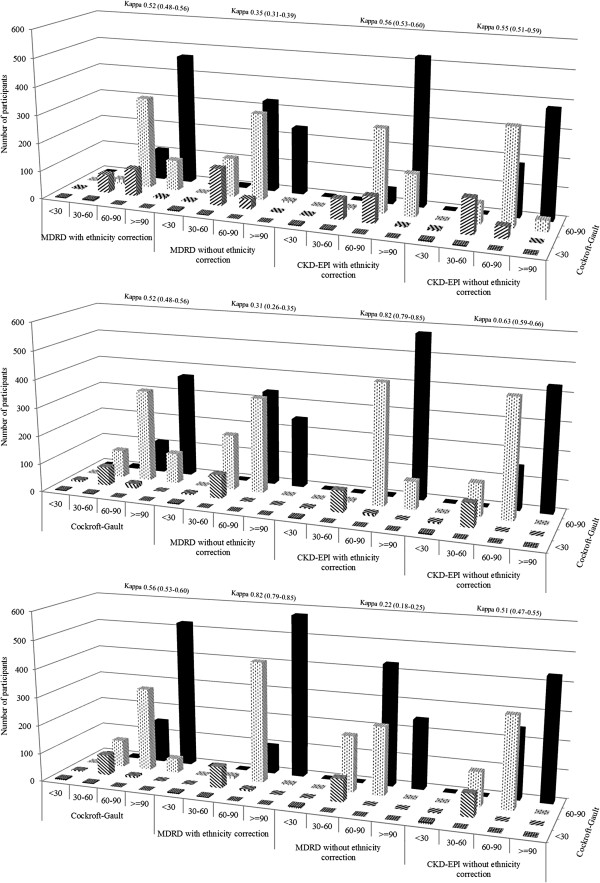
**Chronic kidney disease (CKD) staging and cross classification of participants into 4 eGFR categories (eGFR ≥90 ml/min, 60–90 ml/min, 30–60 ml/min and <30 ml/min) using the Cockcroft-Gault (CG), 4-variable Modification of Renal Disease (4-v-MDRD) and Chronic Kidney Disease Epidemiology Collaboration (CKD-EPI) equations.** The three equations were compared to each other in classifying the study population into CKD stages using Cohen’s kappa statistics, and the kappa index for each comparison is indicated.

None of the participants had an eGFR value <15 ml/min based on any of the estimators. Therefore, kidney function staging was based on 4 categories: ≥90 ml/min, 60–90 ml/min, 30–60 ml/min, and <30 ml/min. There was a substantial variation in the number of participants ranked within kidney function strata by different estimators (Figure 2). The highest agreement was observed when comparing the ranking based on the MDRD with that based on CKD-EPI equations (both with ethnicity correction) with a kappa statistic (95% confidence interval) of 0.82 (0.79-0.85). Among participants ranked according to the MDRD formula without ethnicity correction, the CKD-EPI equation with ethnicity correction always reclassified more participants within more favourable than within less strata of kidney function (Figure 2). For instance, among those with an eGFR of 30–60 ml/min based on MDRD with correction (n=88) the CKD-EPI with correction reclassified 9 (10%) in the stratum 60–90 ml/min and only 3 (3%) in the stratum <30 ml/min. Equivalent results for those within the MDRD stratum 60–90 ml/min (n=531) were 98 (18.4%) and 6 (1.1%). Similar patterns were observed when comparing MDRD and CKD-EPI equivalents without correction for ethnicity, with however a much higher proportions of participants being reclassified, resulting in only average agreement [kappa 0.63 (0.59-0.67)]. The agreement was always lower than average when comparing the Cockcroft-Gault equation with any of the other estimators, with kappa statistic ranging from 0.35 (Cockcroft-Gault vs. MDRD without correction) to 0.56 (Cockcroft-Gault vs. CKD-EPI with correction); Figure 2.

### Crude and age-adjusted prevalence of CKD stage 3–5 and agreement between estimators

**Figure 3:**
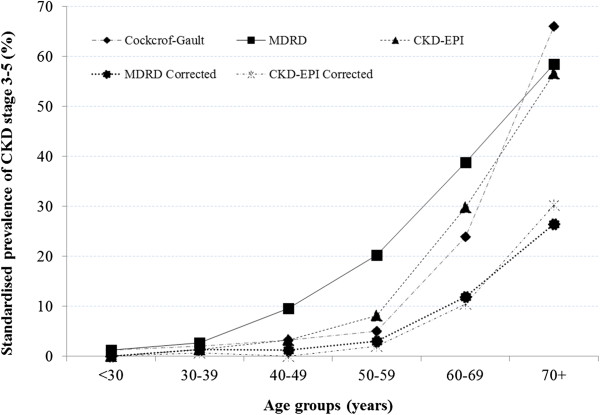
Age-standardised prevalence of CKD stage 3–5 according to GFR estimating equations Cockcroft-Gault 4-variable Modification of Renal Disease (4-v-MDRD) and Chronic Kidney Disease Epidemiology Collaboration (CKD-EPI) equations.

The crude prevalence of CKD stages 3–5 is shown in Table 1 for the total population and by sex. The highest overall prevalence of 26.7%) was observed in women with the MDRD equation without ethnicity correction, and with the Cockcoft-Gault equation in men (19.2%). Generally, CKD stages 3–5 were more prevalent in women except for the the Cockcoft-Gault equation (p=0.014) (Table 1). The age-standardised prevalence (95% confidence interval) of CKD stage 3–5 was 7.2% (5.8-8.6) for Cockcroft-Gault, 3.9% (3.1-4.7) and 13.0% (11.2-14.8) respectively for the MDRD with and without ethnicity correction, and 3.5% (2.7-4.3) and 8.7% (7.5-9.9) for the CKD-EPI equations with and without ethnicity correction. Regardless of the estimator, the prevalence of CKD increased with advancing age, with an accelerated pattern after 50 years of age (Figure 3).

The highest agreement between GFR estimators to diagnosed CKD stage 3–5 was observed when comparing the MDRD and CKD-EPI equations, both with ethnicity correction, kappa 0.91 (95% confidence interval: 0.86-0.95), followed by their equivalents without correction, kappa 0.79 (0.74-0.83) (Table 2). This also applied to a large extent to men and women considered separately. The agreement for other paired comparisons was low to average. With two exceptions, the confidence interval around kappa estimates in men and women always overlapped, suggesting that estimates were not appreciably different by sex.

**Table 2:** Agreement between different kidney function estimators in diagnosing CKD stage 3–5 (kappa statistic and 95% confidence interval)

**Estimator 1**	**Estimator 2**	**Men**	**Women**	**Total**
Cockcroft-Gault	MDRD corrected	0.27 (0.13-0.40)	0.60 (0.52-0.68)	0.51 (0.44-0.58)
	MDRD	0.57 (0.44-0.70)	0.52 (0.45-0.58)	0.53 (0.47-0.59)
	CKD-EPI corrected	0.35 (0.20-0.47)	0.66 (0.58-0.74)	0.57 (0.49-0.64)
	CKD-EPI	0.64 (0.52-0.76)	0.65 (0.59-0.72)	0.65 (0.59-0.71)
MDRD corrected	MDRD	0.46 (0.30-0.60)	0.41 (0.34-0.47)	0.42 (0.36-0.48)
	CKD-EPI corrected	0.87 (0.71-0.97)	0.92 (0.87-0.96)	0.91 (0.86-0.95)
	CKD-EPI	0.53 (0.34-0.70)	0.57 (0.49-0.65)	0.57 (0.50-0.64)
MDRD	CKD-EPI corrected	0.51 (0.35-0.64)	0.38 (0.32-0.45)	0.41 (0.34-0.47
	CKD-EPI	0.85 (0.74-0.93)	0.78 (0.72-0.82)	0.79 (0.74-0.83)
CKD-EPI corrected	CKD-EPI	0.58 (0.43-0.73)	0.54 (0.46-0.61)	0.55 (0.48-0.62)

CG, Cockcroft-Gault; CKD-EPI, Chronic Kidney Disease Collaboration Epidemiology; DBP, 4-v-MDRD, four variable Modification of Diet in Renal Disease.

### Determinants of chronic kidney disease, stages 3–5

Age and sex adjusted determinants of prevalent CKD stage 3–5 are shown in Table 3, separately for each of the estimators. In multivariable logistic regression models with mutual adjustment for significant determinants in sex and age adjusted models, sex, age and known hypertension were consistently associated with CKD stage 3–5 across the 5 estimators (Table 4) with similar range of effects. Furthermore triglycerides was associated with CKD based on ethnicity corrected MDRD or CKD-EPI equations, while body mass index was associated with Cockroft-Gault formulae defined CKD stage 3–5.

**Table 3:** Determinants of CKD stage 3–5 (age, sex and cohort adjusted analyses)

**Variables**	**MDRD**	**MDRD corrected**	**CKD-EPI**	**CKD-EPI corrected**	**CG**
Age (/year)	1.10 (1.08-1.11)	1.11 (1.09-1.13)	1.13 (1.11-1.15)	1.14 (1.11-1.17)	1.18 (1.15-1.21)
Female gender	3.08 (2.07-4.69)	2.42 (1.34-4.63)	2.56 (1.63-4.10)	2.09 (1.15-3.98)	0.74 (0.47-1.63)
Cohort	0.24 (0.15-0.39)	0.44 (0.19-0.91)	0.34 (0.19-0.58)	0.28 (0.09-0.66)	0.35 (0.18-0.64)
Current smoking	0.99 (0.71-1.39)	0.67 (0.37-1.17)	0.84 (0.55-1.26)	0.55 (0.38-1.02)	1.72 (1.09-2.77)
BMI (/kg/m^2^)	1.01 (0.99-1.03)	1.01 (0.98-1.05)	1.01 (0.98-1.04)	1.00 (0.96-1.04)	0.82 (0.79-0.86)
WC (/cm)	1.00 (0.99-1.01)	1.01 (1.00-1.03)	1.00 (0.99-1.02)	1.01 (0.99-1.02)	0.93 (0.91-0.94)
Hip circumference (/cm)	1.00 (0.99-1.02)	1.00 (0.99-1.02)	1.00 (0.99-1.02)	1.00 (0.98-1.02)	0.91 (0.89-0.93)
WHR (/unit)	0.66 (0.09-4.68)	14.63 (0.89-152)	1.01 (0.10-9.85)	9.86 (0.40-176)	0.03 (0.002-0.44)
SBP (/mm Hg)	1.00 (0.99-1.01)	0.99 (0.98-1.01)	1.00 (0.99-1.01)	0.99 (0.98-1.00)	0.99 (0.98-1.00)
DBP (/mm Hg)	1.00 (0.99-1.02)	1.00 (0.98-1.02)	1.00 (0.99-1.02)	1.00 (0.98-1.01)	0.98 (0.96-1.00)
Known hypertension	1.96 (1.35-2.90)	2.54 (1.35-5.16)	1.98 (1.27-3.18)	2.55 (1.31-5.31)	1.34 (0.82-2.23)
FBG (/mmol/L)	0.98 (0.93-1.03)	1.06 (0.99-1.13)	1.00 (0.94-1.06)	1.05 (0.97-1.13)	0.97 (0.90-1.04)
PostBG (/mmol/L)	0.97 (0.92-1.02)	0.96 (0.88-1.03)	0.96 (0.90-1.01)	0.95 (0.87-1.03)	0.93 (0.87-1.00)
HbA1c (/%)	0.95 (0.84-1.06)	1.16 (1.00-1.32)	1.00 (0.88-1.13)	1.15 (0.98-1.33)	1.00 (0.86-1.15)
TC (/mmol/L)	1.10 (0.96-1.26)	1.04 (0.85-1.27)	1.19 (1.02-1.39)	0.96 (0.78-1.18)	0.96 (0.80-1.15)
TG (/mmol/L)	1.13 (0.96-1.33)	1.37 (1.07-1.70)	1.23 (1.01-1.48)	1.37 (1.04-1.75)	0.97 (0.74-1.25)
HDL-C (/mmol/L)	0.88 (0.55-1.39)	0.44 (0.20-0.92)	0.95 (0.55-1.61)	0.54 (0.24-1.16)	2.12 (1.19-3.74)
LDL-C (/mmol/L)	1.09 (0.94-1.27)	1.02 (0.81-1.28)	1.17 (0.98-1.39)	0.92 (0.72-1.16)	0.88 (0.72-1.07)
Serum cotinine	0.95 (0.86-1.05)	0.93 (0.79-1.08)	0.95 (0.85-1.07)	0.90 (0.76-1.07)	1.22 (1.07-1.39)
GGT (/unit)	1.09 (0.86-1.38)	1.17 (0.79-1.72)	1.03 (0.76-1.38)	1.20 (0.78-1.83)	1.25 (0.88-1.76)
Any diabetes	1.17 (0.83-1.63)	1.55 (0.96-2.46)	1.16 (0.79-1.69)	1.55 (0.94-2.55)	1.12 (0.73-1.71)

***Abbreviations*:** BMI, body mass index; CG, Cockcroft-Gault; CKD-EPI, Chronic Kidney Disease Collaboration Epidemiology; DBP, diastolic blood pressure; FBG, fasting blood glucose; GGT, gamma glutamyltransferase; HbA1c, glycosylated haemoglobin A1c; HDL-C, high density lipoprotein-cholesterol; LDL-C, low density lipoprotein-cholesterol; 4-v-MDRD, four variable Modification of Diet in Renal Disease; SBP, systolic blood pressure; TC, total cholesterol; TG, triglycerides; WHR, waist-to-hip ratio.

**Table 4:** Multivariable adjusted odd ratios (and 95% confidence intervals) for the determinants of CKD stage 3-5

**Variables**	**MDRD**	**MDRD corrected**	**CKD-EPI**	**CKD-EPI corrected**	**Cockroft-Gault**
Age (/year)	1.09 (1.08-1.11)	1.11 (1.09-1.14)	1.14 (1.11-1.15)	1.14 (1.11-1.17)	1.21 (1.18-1.25)
Female gender	2.88 (1.92-4.38)	2.15 (1.18-4.14)	2.15 (1.36-3.47)	1.97 (1.07-3.83)	1.43 (0.84-2.45)
Cohort	0.27 (0.16-0.44)	0.50 (0.21-1.04)	0.39 (0.22-0.68)	0.31 (0.10-0.73)	0.30 (0.14-0.60)
Known hypertension	1.96 (1.35-2.90)	2.42 (1.28-4.94)	1.97 (1.25-3.15)	2.34 (1.20-4.93)	2.76 (1.56-5.00)
Triglycerides (/mmol/L)	NS	1.33 (1.04-1.67)	NS	1.33 (1.00-1.71)	NS
Body mass index (/kg/m^2^)	NS	NS	NS	NS	0.81 (0.77-0.85)
Total cholesterol (/mmol/L)	NS	NS	1.19 (1.02-1.39)	NS	NS

CKD-EPI, Chronic Kidney Disease Collaboration Epidemiology; 4-v-MDRD, four variable Modification of Diet in Renal Disease.

For CKD-EPI, TC was only significant when triglycerides (NS) was removed from the model, however triglycerides in the presence of other covariates except total cholesterol was not significant. For Cockroft-Gault: Hip was significant when replacing waist circumference in the models, not in its presence. And hypertension was significant only in the presence of body mass index in the model.

## Discussion

The present study investigated the prevalence of CKD using, MDRD, CKD-EPI and the Cockcroft-Gault equations. We obtained different estimates of GFR by the three commonly used CKD equations, resulting in varying prevalence rates of CKD in the mixed ancestry population from South Africa. The highest agreement was observed when comparing the MDRD and CKD-EPI equations, both with ethnicity correction, followed by their equivalents without correction. Based on the South African Renal Society CKD guidelines that omit the correction factor except for black Africans, the prevalence of CKD stages greater than 3 is the highest reported in Africa thus far. In this first community-based study on the prevalence of CKD in an urban population from South Africa, regardless of the estimator, the prevalence of CKD increased with advancing age, with an accelerated pattern after 50 years of age. Risk factors for CKD such as sex, age and hypertension were similar to those reported in developed countries.

To our knowledge, there exist only two detailed reports on the prevalence of CKD in populations from Sub-Saharan Africa [9,11]. Both studies reported prevalence rates much lower than obtained in this study using either MDRD and/or CKD-EPI equations, but higher with Cockcroft-Gault [11]. Similar to other population studies, majority of our participants with eGFR < 60 ml/min were stage 3 and none were stage 5 (eGFR < 15 ml/min) [11,28,29]. Progression of CKD to ESRD is reported to be at a slower rate in women [30], but we found a higher CKD prevalence in females than in males similar to other epidemiological CKD screening studies [31-33] though some studies have reported higher rates in men [9,28]. Gender-specific differences in glomerular structure, hemodynamic condition and the effect of sex hormones on kidney cells are some of the factors believed to contribute to gender differences [34]. In our study most women were probably postmenopausal as their mean age was 52.4 years and might therefore explain the divergent findings.

The Cockcroft-Gault, MDRD and CKD-EPI equations have been adopted and used globally as cost-effective methods for CKD diagnosis and kidney function staging; and these equations have been validated in a few African studies [9-12]. Currently in South Africa, the MDRD and Cockcroft-Gault formulae are the recommended methods for the estimation of GFR, but we observed a poor agreement between these two equations. Instead the agreements were always higher between the MDRD and CKD-EPI equations. Whilst the South African, Renal Society CKD guidelines recommend the MDRD with ethnicity correction factor only in black Africans, it does not account for the African ancestry in the mixed ancestry population of South Africa. Nevertheless, evaluation of eGFR equations against creatinine clearance involving individuals from Africa have shown that the MDRD and CKD-EPI formulae performed better when the African-American-derived ethnicity correction factor was excluded [10-12]. Although we demonstrated the best agreement and similar CKD rates with the MDRD and CKD-EPI equations with the ethnicity correction factor, further studies are required to evaluate these equations against the gold standard in this mixed ancestry population of South Africa.

This study also adds to already existing evidence that hypertension is one of the major contributors to the development and/or progression of CKD in South Africa and worldwide [12,35,36]. In our population the presence of hypertension doubled the risk of prevalent CKD. Hypertension affects approximately 25% of the adult population and is a cause of CKD in 21% of patients on renal replacement therapy in the South African Registry [37]. CKD is also a major contributor of secondary dyslipidaemia and is characterized by specific abnormalities involving all the lipoprotein classes, with variations depending on the degree of renal impairment, etiology of primary renal disease and the method of dialysis [38]. However, in this study, triglycerides were only associated with CKD based on ethnicity corrected MDRD or CKD-EPI equation, and only in the absence of other covariates in logistic regression models.

Our study has some limitations. This study was conducted at only one geographical site, and may not adequately reflect all the mixed ancestry population groups in Sub-Saharan Africa. The Cockcoft-Gault, MDRD and CKD-EPI equations were not evaluated against gold standard to assess their validity in estimating GFR in this population. We did not have estimates of albuminuria which are required for clinical and aetiological diagnosis of CKD in clinical settings. This additional information is important particularly in the interpretation of eGFR > 60 where inaccuracies of the eGFR equations are greatest. The nature of this study is cross-sectional with high female to male participation, the latter being a common trend in South African population studies. Our study also has major strengths including the provision of a complete picture of the CKD prevalence in a South African community setting with a high prevalence of obesity, diabetes and hypertension. The importance of assessing the GFR prediction equations in specific ethnic population groups before they can be recommended for use in each country has been highlighted. Our study provides evidence against uncritical recommendation of GFR estimators in this population, as this may result in misclassification of individuals risk status and inappropriate health actions.

## Conclusion

The findings of the present study have significant clinical and public health implications. We studied a community with a high burden of obesity, hypertension and diabetes that may also be at increased risk of CKD. It is highly likely that some of the participants identified to be at risk or have CKD in the present study were not aware of their status as this was a community-based research project. Such individuals should be monitored to delay or prevent the progression of the diseases. The South African Renal Society CKD guidelines recommends the use of Cockcroft-Gault or MDRD equations, however, our study demonstrated a poor agreement between the two equations in the Mixed Ancestry population groups. Other African studies have suggested omission of the ethnicity correction factor from either the MDRD or CKD-EPI equation. Our study found a high positive correlation and good agreement when the ethnicity factor was included. The findings of the present study suggest that the Cockcoft-Gault equation be replaced by the CKD-EPI equation upon validation against measured GFR to prevent overestimation of CKD, particularly in a country with limited health care resources. Identification of an eGFR equation that can be used in primary or secondary healthcare institution may lead to the introduction of automated eGFR reporting, and better management of the disease. Studies in the United Kingdom and Australia have shown that automated reporting of eGFR with serum creatinine increased primary care provider awareness of CKD [39,40], and in other countries nephrology visits [41].

## Abbreviations

CKD: Chronic kidney disease; KDOQI: Kidney disease outcomes quality initiative; ESRD: End stage renal disease; CVD: Cardiovascular diseases; GFR: Glomerular filtration rate; SSA: Sub-Saharan Africa; MDRD: Modification of diet in renal disease; CKD-EPI: CKD Epidemiology collaboration; WHO: World health organisation; OGTT: Oral glucose tolerance test; HbA1c: Glycated haemoglobin; SD: Standard deviation.

## Competing interests

The authors do not have any competing interests.

## Authors’ contributions

TEM: Conception, design, interpretation of data, drafting, revising the article and final approval of the version to be published. YYY: Drafting, revising the article and final approval of the version to be published. MAR: Drafting, revising the article and final approval of the version to be published. MSH: Revising the article and final approval of the version to be published. APK: design, analysis and interpretation of data, revising the article and final approval of the version to be published. RTE: Conception, revising the article and final approval of the version to be published. All authors read and approved the final manuscript.

## Pre-publication history

The pre-publication history for this paper can be accessed here:

http://www.biomedcentral.com/1471-2369/14/75/prepub
